# Sex Differences in Voyeuristic and Exhibitionistic Interests: Exploring the Mediating Roles of Sociosexuality and Sexual Compulsivity from an Evolutionary Perspective

**DOI:** 10.1007/s10508-021-01991-0

**Published:** 2021-07-06

**Authors:** Andrew George Thomas, Bridie Stone, Paul Bennett, Steve Stewart-Williams, Leif Edward Ottesen Kennair

**Affiliations:** 1 Department of Psychology, Swansea University, Swansea, SA2 8PP UK; 2 School of Psychology, University of Nottingham Malaysia, Semenyih, Malaysia; 3 Department of Psychology, Norwegian University of Science and Technology, Trondheim, Norway

**Keywords:** Voyeurism, Exhibitionism, Evolutionary psychology, Sociosexuality, Sex differences, Mating strategies

## Abstract

Sociosexuality and sexual compulsivity predict sex differences in voyeuristic interest in the population. In this study, we used a sample of 1113 participants from the UK (46% men) to consider whether sociosexuality and sexual compulsivity interacted to explain these sex differences and whether this relationship extended to the related domain of exhibitionism. In doing so, we tested novel predictions derived from an evolutionary perspective which views voyeuristic and exhibitionistic interest as manifestations of a short-term mating strategy. Participants reported their levels of repulsion toward voyeurism and exhibitionism and their interest in performing such acts under different levels of risk. There were clear sex differences in voyeuristic and exhibitionistic repulsion that were partially mediated by the serial combination of sociosexuality and sexual compulsivity. Examining the sexes separately revealed qualitatively different relationships between sociosexuality and sexual compulsivity when predicting exhibitionistic, but not voyeuristic, repulsion. Combined, sociosexuality and sexual compulsivity also mediated the sex difference in willingness to commit acts of voyeurism, but not exhibitionism, which was equally low for both sexes. The results highlight the role sociosexuality plays in voyeuristic and exhibitionistic interest, which coupled with an evolutionary perspective, may have implications for how we view courtship disorders.

## Introduction

Courtship disorders, such as voyeurism and exhibitionism, represent a well-studied subset of paraphilia associated with the early stage of the courtship process. Like other paraphilic disorders, they are often considered “male disorders” because they are predominantly diagnosed in men rather than women (Beech et al., 2016; Thibaut et al., 2016). In voyeurism, arousal comes from watching others undress or engage in sexual activities, a disturbance in the search phase of courtship. In exhibitionism, arousal comes from exposing oneself to strangers, a disturbance in the pre-tactical phase (Freund, 1990). To meet the criteria for paraphilic disorder, perpetrators must involve nonconsenting persons or feel distressed by their own actions (American Psychiatric Association, 2013). Such diagnoses are rare (Beech et al., 2016); however, one also finds interest in these behaviors among the general population, at the level of fantasy, arousal, and interest in committing such acts. Explaining variation in this interest within nonclinical samples may help us understand the stark sex differences at the disorder level.

### Exhibitionistic and Voyeuristic Interests

By some estimates, 35–47% of the population have an interest in performing an act of voyeurism (Ahlers et al., 2011; Joyal & Carpentier, 2017). Estimates for exhibitionism in its strict sense (exposure of genitals to a stranger) are much lower, at around 4%, but this rises to 31% when extended to include related acts (e.g., having sex in a place where a stranger might see; Joyal & Carpentier, 2017). These interests have been examined in relation to a range of individual and contextual factors including sex drive and hypersexuality (Bouchard et al., 2017; Dawson et al., 2016; Långström & Hanson, 2006), physical, sexual, and mental health (Långström & Seto, 2006; Oliveira Júnior & Abdo, 2010), sociosexuality (Dawson et al., 2016), and the level of risk involved (Rye & Meaney, 2007). Culturally diverse samples suggest that these interests are not restricted to Western cultures (Bhugra et al., 2010; Makanjuola et al., 2008).

Importantly, there are sex differences in interest. Men report feeling less repulsed by the idea of voyeurism than women do (*d* = 0.66) and show a greater willingness to engage in it (*d* = 0.52; Dawson et al., 2016; Joyal & Carpentier, 2017). Similar, though smaller, differences can be found for exhibitionism (*d* = 0.39 and 0.18, respectively).^1^ Research using nonclinical samples finds that sex differences in these interests are mediated by traits such as sociosexuality and sexual compulsivity. For example, a recent study investigated some of the predictors of paraphilic interest in a large nonclinical sample from Canada (Dawson et al., 2016). Sex differences were found for most interests, with men finding them broadly less aversive than women (*d* = 0.40). A series of mediation models revealed that sex differences in general paraphilic interests were mediated by a number of traits, including sex drive—a composite of three different measures of sexual preoccupation of which sexual compulsion was the strongest predictor. In the specific case of voyeuristic repulsion (the largest sex difference; *d* = 0.66), sociosexuality and sex drive were the only two variables that partially mediated this difference.^2^

Four questions arise from extant research that prompt a more in-depth investigation of the role of sociosexuality and sexual compulsivity. First, how might sociosexuality and sex drive interact when mediating sex differences? To answer this question, we apply an evolutionary lens to the role of sociosexuality in voyeuristic and exhibitionistic interest. Evolutionary theory is a powerful tool for explaining sex differences across many domains of mating behavior (Buss & Schmitt, 1993; Stewart-Williams & Thomas, 2013b; Thomas, 2018) and so seems well placed to explain such differences in interests and behaviors relating to the early stage of courtship. Specifically, it generates novel predictions about the relationship between predictors/mediators of voyeuristic and exhibitionistic interest. Historically, these traits have been examined using separate models (e.g., Bouchard et al., 2017; Dawson et al., 2016) meaning that the reported effects do not account for shared variance. Indeed, these traits correlate fairly strongly (Ostovich & Sabini, 2004), which casts doubt on their influence being orthogonal. As discussed later, an evolutionary perspective can also provide novel insights when trying to understand the origins of extreme nonfunctional behaviors, such as courtship disorders.

Second, does the explanatory power of sociosexuality and sex drive also extend to exhibitionistic interests? These traits have been shown to mediate sex differences in general paraphilic and, specifically, voyeuristic arousal (Bouchard et al., 2017; Dawson et al., 2016). We ask whether sex differences in exhibitionistic interest can be mediated in the same way. *Prima facie*, these paraphilic interests seem to have conceptual overlap. They are both issues of “courtship” that involve precursors to sexual activity with an unknown individual. They also correlate strongly (*r* ~ .50; Dawson et al., 2016) and show comorbidity at the disorder level (Freund, 1990).

Third, are the underpinnings of voyeuristic/exhibitionistic interest sex-specific? Controlling for one psycho-biologically sex-linked trait (e.g., sociosexuality) while studying another may reduce sex differences, but it also removes the ability to examine other forms of sexual differentiation, such as unique within-sex predictive patterns (Kennair et al., 2016). Indeed, attempts to explain away sex differences in this manner exist in the literature (e.g., Conley, 2011). Therefore, Kennair and Bendixen (2018) suggest that a better analytical strategy is to run separate analyses for each sex to consider whether predictive patterns are sexually differentiated. In the case of sociosexuality, sex-specific analysis can reveal the effect of masculinization on the sexual psychology of each sex. As we have no a priori predictions for the nature of sexual differentiation in this research, we adopt the default position that the underlying relationships for each sex will be similar.

Finally, do the mediation effects of sex drive and sociosexuality extend beyond arousal/repulsion and into anticipated action? Rye and Meaney (2007) found that participants were more willing to commit a hypothetical act of voyeurism when the risk of getting caught was negligible. While they reported no sex difference in willingness to commit a voyeuristic act, a closer inspection of the percentage of participants who indicated they would be extremely likely to commit an act of voyeurism reveals that twice as many men selected this option than women (44% vs 22%, when correcting for row totals). While the study itself was underpowered (smallest cell *n* = 13), the methodology has its merits. Combined with a within-subjects design (see Stewart-Williams et al., 2017), it would allow us to examine (a) how sex differences change when shifting focus from aversion to (anticipated) action within the same sample; and (b) whether controlling for sociosexuality and sexual compulsivity reduces this difference in both domains.

### Sociosexuality as a Measure of Evolved Mating Strategy

When used as a measure of individual differences, sociosexuality is occasionally divorced from its evolutionary underpinnings (e.g., Hall & Pichon, 2014; Weiser et al., 2018). However, doing so can often lead to theoretically barren explanations for behavior. Within evolutionary psychology, sociosexuality is not just seen as a measure of desire and willingness to have uncommitted sex, but as a proxy of mating strategy—suites of evolved psychological mechanisms which facilitate short- and long-term mating in ways that would have historically enhanced fitness (Buss & Schmitt, 1993; Gangestad & Simpson, 2000). An individual’s mating strategy calibrates their mating interests and preferences (Li & Kenrick, 2006; Stewart-Williams et al., 2017; Thomas, 2018) including frequent and intense desires to have sex in the absence of commitment (Penke & Asendorpf, 2008). Because the sexes are asymmetrical in their levels of obligatory parental investment and their ability to enhance fitness directly by having sex with multiple partners, men have evolved a greater inclination toward adopting a short-term mating strategy than women (Stewart-Williams & Thomas, 2013b; Thomas, 2018).

This evolutionary framework can help explain why sociosexuality is associated with voyeuristic and exhibitionistic interest. Short-term strategies have evolved in response to the adaptive problems associated with that form of mating—problems such as identifying and capitalizing on sexual access rather than assessing a mate’s compatibility as a potentially pair-bonded partner and co-parent (Buss & Schmitt, 1993; Howell et al., 2012; Treger & Sprecher, 2011). Thus, we may expect that modern humans employ a short-term mating strategy to be drawn toward situations which may facilitate spontaneous and opportunistic mating with strangers—in the present case, an interest is in observing others undress, engage in sex, or exposing oneself to others. Both voyeuristic and exhibitionistic desires share a common root in their spontaneous and opportunistic approach to the early stage of courtship (Freund, 1990). While it is feasible that they may have been subjected to different selection pressures, we make no predictions about this in the current work, expecting instead to find that the relationship between these desires and mating strategy to be qualitatively similar.

This perspective provides several insights relevant to the current work. First, it suggests that the relationship between sociosexuality and sexual compulsivity should be hierarchical rather than orthogonal; sexual compulsivity should be driven by mating strategy. Previous research has not tested this serial relationship, instead focusing on the use of multiple mediation models (e.g., Dawson et al., 2016). Second, it suggests that sociosexuality and sexual compulsivity should explain voyeuristic and exhibitionistic repulsion in similar ways. Finally, it suggests that disorders may represent abnormal “nonfunctional” levels of interest in these activities. This may also explain the marked sex difference in the prevalence of voyeuristic and exhibitionistic disorders. Because group differences at the mean tend to translate to larger group differences at the extremes (Stewart-Williams & Thomas, 2013a), it follows that men will represent a larger proportion of those exhibiting an extreme version of a short-term mating strategy and its accompanying desires and behaviors.

### Hypotheses

In the present study, we used a large nonclinical sample to examine voyeuristic and exhibitionistic repulsion and anticipated action. Based on the outstanding questions above, we had five hypotheses:

**H1:** Men will report less voyeuristic and exhibitionistic repulsion than women.

**H2:** A serial combination of sociosexuality and sexual compulsivity will mediate this sex difference.

**H3:** Sociosexuality and sexual compulsivity will predict voyeuristic and exhibitionistic repulsion within each sex in similar ways.

**H4:** Men will be more willing to commit acts of exhibitionism and voyeurism than women.

**H5:** A combination of sociosexuality and sexual compulsivity will also mediate these sex differences.

## Method

### Participants

Participants were invited to complete a study examining “individual differences in sexual behavior, attitudes, and preferences [and how these relate to] preferences for long-term or short-term relationships.” The vast majority of the participants were recruited online, with a small percentage (< 5%) recruited from Swansea University’s participant pool in exchange for course credit. As correlations stabilize at *N* = 250 (Schönbrodt & Perugini, 2013), our original target was to recruit 250 men and 250 women to allow for sex-specific relationships to be explored. However, particularly effective snowball sampling meant that we ended up with a sample that greatly exceeded this.

To be included in the final analyses, participants had to be over 18 years and regard their sex as male or female. Due to the sensitive nature of the questions, it was possible that the participants’ answers did not reflect their actual interests and behaviors. To allow participants to indicate that their answers may not be reliable, at the end of the study they rated their truthfulness on a 7-point Likert scale ranging from 1 (*I was not truthful at all*) to 7 (*I answered every question truthfully*). Those participants who answered with a score of 4 or less (*n* = 15) were removed from the analyses.^3^

The final sample consists of 1,113 participants ranging from 18 to 70 years old (*M* = 26.11, *SD* = 8.13), 46% of which were male. The sample was predominantly White (91%), childless (82%) and described their social status as either middle (42%) or lower-middle class (32%). The bulk of the sample were heterosexual (79%) with bisexuality (12%) and homosexuality (7%) as the second and third most common sexualities. Finally, the sample primarily consisted of those in a committed relationship (61%) and those single, separated, or in an uncommitted relationship (37%).

### Measures

Sociosexuality was measured using the Sociosexual Orientation Inventory-Revised (SOI-R; Penke & Asendorpf, 2008). This inventory includes nine items measuring one’s willingness to have uncommitted sex based on one’s behaviors, attitudes, and desires. Example questions include “With how many different partners have you had sexual intercourse without having an interest in a long-term committed relationship with this person?” and “I can imagine myself being comfortable and enjoying "casual" sex with different partners.” Each question is answered using a 9-point scale with varying labels/anchors. A total score is calculated by averaging across questions. The scale proved reliable for our study with *α* = 0.87. High scores indicate that an individual favors sex in the absence of commitment, while low scores indicate a preference for sex as part of a committed relationship. Sexual compulsivity was measured using the Sexual Compulsivity Scale (SCS; Kalichman et al., 1994). This scale includes ten items assessing the extent to which an individual’s sexual thoughts and behaviors interfere with their daily life. Items include “I have to struggle to control my sexual thoughts and behavior” and “I think about sex more than I would like to.” Each item is responded to using a 4-point Likert scale ranging from 1 (*Not at all like me*) to 4 (*Very much like me*). A total is calculated by averaging the scores from each question with higher totals representing greater sexual compulsivity. This scale was also reliable (*α* = 0.87).

Paraphilic repulsion was measured using the Paraphilia Scale (Seto et al., 2012). The scale includes 40 statements that depict sexual thoughts or activities, 32 of which relate to paraphilia. These include “You are watching an unsuspecting stranger while they undress” and “You are exposing your penis to a stranger who is not expecting it.” For each statement, participants use a 7-point Likert scale ranging from -3 (*Very Repulsive*) to + 3 (*Very Arousing*) to respond to the question “Please rate how sexually arousing or sexually repulsive you currently find each of the following activities, whether you have tried it or not.” A total score is calculated by averaging the responses to each paraphilia statement, with higher scores representing greater paraphilic interest. The example items above capture voyeuristic and exhibitionistic arousal, respectively. For the purpose of our study, we changed “penis” in the exhibitionism statement to “genitals.”

Finally, willingness to engage in voyeuristic or exhibitionistic acts was measured using hypothetical vignettes adapted from Rye and Meaney (2007). For voyeurism, participants are given the scenario “You see someone who you find very attractive. The person does not suspect that you can see him or her. He/she begins undressing.” They are then asked to rate their likelihood of watching the person undress under six different levels of risk using a 7-point Likert scale from -3 (*Very Unlikely*) to + 3 (*Very likely*). The levels of risk were illustrated via the % chance of getting caught–0%, 10%, 25%, 50%, 75%, and 100%. The exhibitionism scenario was as follows: “You see a man/woman who you find very attractive. How likely would it be that you would expose your private areas such as genitals, breasts, or buttocks, to them from a distance?” Each scenario was tailored to the participant’s sex and sexual orientation. For example, scenarios given to heterosexual or bisexual men had female targets, whereas those seen by homosexual men had male targets.

### Procedure

The study was conducted online via Qualtrics. After reading an information sheet and providing informed consent, participants completed the measures and tasks in the order presented in the Measures section. Before completing the SCS, paraphilia scale, and the hypothetical scenario task, participants were given a warning of the sensitive nature of the questions and reminded that they were free to withdraw from the study at any time by closing the browser window. After completing all sections, participants were asked about their truthfulness and then presented with a debrief screen.

## Results

**Fig. 1:**
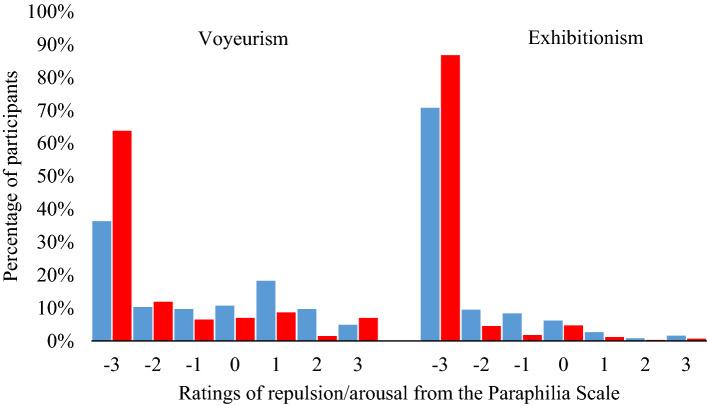
Distribution of responses to the repulsion/arousal questions on the Seto et al. (2012) Paraphilia Scale related to voyeurism (left) and exhibitionism (right). The responses of men are shown in blue and women in red

As predicted, sex differences were present for both the voyeurism and exhibitionism items of the Paraphilia Scale with men showing greater interest than women. Not surprisingly, floor effects were present for both variables (see Fig. 1). For voyeurism, 63.6% of men selected a value other than -3 compared to 36.2% of women. A bootstrapped *t* test (*n* = 1,000) revealed a mean difference of 1.21 (95% CI = 1.00, 1.43) between the sexes (*t*[1111] = 11.52, *p* < .001. This difference constituted a large effect of *d* = 0.69 (CI = 0.57, 0.82), likely to hold explanatory and practical significance in both single events and over time (Funder & Ozer, 2019). For exhibitionism, 29.2% of men selected a value other than -3 compared to 13.2% of women. Analysis revealed a mean difference of 0.37 (CI = 0.23, 0.50) between the sexes (*t*[1111] = 5.33, *p* < .001). This difference constituted a small-to-medium effect of *d* = 0.32 (CI = 0.21, 0.44) indicating that this difference may be small in terms of single events but potentially holds explanatory significance over time (Funder & Ozer, 2019). Thus, we found support for H1, which predicted a sex difference in self-reported repulsion for both activities. Further analyses revealed that voyeuristic and exhibitionistic repulsion correlated positively with sociosexuality and sexual compulsivity, while sex (1 = male, 2 = female) correlated negatively (see Table 1). The strongest relationship was between voyeuristic and exhibitionistic interests (*r* = .56).

**Table 1:** Correlations between voyeuristic and exhibitionistic repulsion and sex, sociosexuality, and sexual compulsivity

Variable	1	2	3	4
1. Voyeurism				
2. Exhibitionism	.56			
	[.51, .60]			
3. Sex [1 = men, 2 = women]	− .33	− .16		
	[− .38, − .27]	[− .22, − .10]		
4. Sociosexuality	.32	.23	− .32	
	[.26, .37]	[.17, .28]	[− .38, − .27]	
5. Sexual compulsion	.34	.28	− .24	.42
	[.28, .40]	[.21, .36]	[− .30, − .18]	[.36, .47]

Values in square brackets indicate bootstrapped (*n* = 1,000) 95% confidence intervals. Low voyeurism and exhibitionism scores reflect higher levels of repulsion. All coefficients have *p* values < .01

**Fig. 2:**
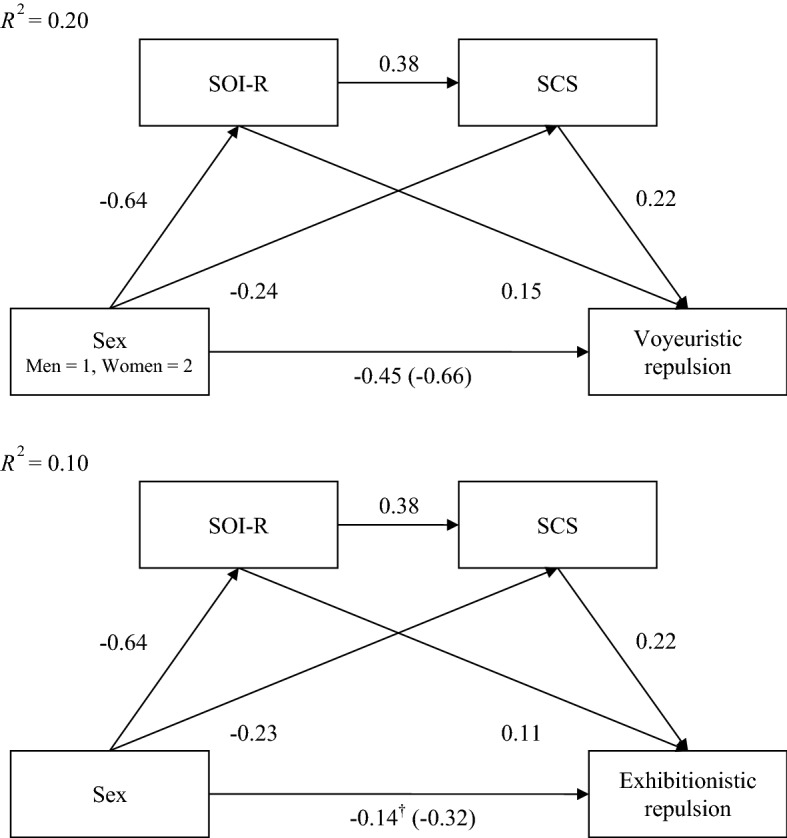
Serial mediation models for sex and **a** voyeuristic and **b** exhibitionistic repulsion via sociosexuality (SOI-R) and sexual compulsivity (SCS). *Note:* Low voyeurism and exhibitionism scores reflect higher levels of repulsion. All betas hold *p *values < .01 with the exception of † (*p* = .02) (Color figure online)

To account for this sex difference, we ran serial mediation models for voyeuristic and exhibitionistic repulsion. Both models used sociosexuality and sexual compulsivity as mediators, and the serial nature of the models allowed us to examine the indirect pathway through sociosexuality and sexual compulsivity, accounting for their theoretical relationship. We found that the influence of sex on voyeuristic repulsion was partially mediated by sociosexuality and sexual compulsivity, with the overall model accounting for 31% of the relationship. As Fig. 2a illustrates, the standardized indirect effect of sex on voyeuristic interest via SOI-R was − .10 (bootstrapped 95% CI = − .14, − .06; sample *n* = 5000) meaning that sociosexuality accounted for roughly 15% of the contribution of sex to voyeuristic repulsion. The indirect effect via sexual compulsivity (− .05; 95% CI = − .08, − .02) accounted for a further 8% of the sex difference. Finally, an indirect effect of sex on voyeuristic repulsion via the serial combination of sociosexuality and compulsivity (− .05; 95% CI = − .07, − .04) accounted for an additional 8%.^4^

A similar model emerged for the relationship between sex and exhibitionistic repulsion (see Fig. 2b) with the overall model accounting for 56% of the relationship. There was a standardized indirect effect of sex on exhibitionistic interest via SOI-R (− .08; CI = − .14, − .03) such that sociosexuality accounted for roughly 23% of the total effect of sex on exhibitionistic repulsion. The indirect effect via sexual compulsivity (− .06; CI = − .10, − .03) accounted for a further 16% of the sex difference. Finally, an indirect effect of sex on exhibitionistic interest via sociosexuality and compulsivity (− .06; CI = − .09, − .03) accounted for an additional 16%. Together, these results provide partial support H2, which predicted that a serial combination of sociosexuality and sexual compulsivity would mediate the sex difference.

### Intrasexual Differences

Examining intergroup differences are just one way of exploring differences between the sexes. Another is to examine whether the relationships between variables shows a sex-differentiated pattern. To see whether sociosexuality and sexual compulsivity predict voyeuristic and exhibitionistic repulsion in sex-specific ways, we ran a series of regression models. All models examined the effect of sociosexuality on repulsion in Step 1 and then included sexual compulsivity in Step 2. As shown in Table 2, sociosexuality was a positive predictor of voyeuristic repulsion in both models, though it was partially mediated by the inclusion of sexual compulsivity. This pattern was qualitatively similar for both sexes, although the contribution of sociosexuality and sexual compulsivity to repulsion was somewhat smaller for women than men.

**Table 2:** Sex-specific regressions for voyeuristic and exhibitionistic repulsion using sociosexuality in Step 1 with the addition of sexual compulsivity in Step 2

	Men		Women	
	Model 1	Model 2	Model 1	Model 2
*Voyeuristic repulsion*				
Sociosexuality	0.33 [0.24, 0.42]	0.22 [0.13, 0.33]	0.19 [0.10, 0.26]	0.11† [0.02, 0.19]
Sexual compulsivity		0.85		0.68
		[0.50, 1.24]		[0.36, 1.00]
adj. *R*^2^	.08	.12	.04	.08
*ΔR*^*2*^		.05		.04
*Exhibitionistic repulsion*				
Sociosexuality	0.17 [0.12, 0.23]	0.13 [0.07, 0.20]	0.09 [0.03, 0.16]	0.02† [− 0.04, 0.08]
Sexual compulsivity		0.32		0.66
		[0.09. 0.57]		[0.32, 1.00]
adj. *R*^2^	.05	.06	.02	.11
Δ*R*^2^		.02		.09

Unstandardized beta values are accompanied by bootstrapped (*n* = 1,000) 95% confidence intervals. Low voyeurism and exhibitionism scores reflect higher levels of repulsion. All coefficients and Δ*R*^2^ hold *p* values < 0.01 with the exception of † (*p* = 0.59)

A more sex-specific pattern was found for exhibitionistic repulsion. For men, this pattern was similar to that of voyeuristic repulsion; sociosexuality was a positive predictor in both models and was partially mediated by compulsivity. However, for women, sexual compulsivity fully mediated the effect of sociosexuality on their reported repulsion. Together, we found only partial support for H3: The pattern of predictors was similar for men and women only in the case of voyeuristic repulsion.

### Anticipated Action Under Varying Levels of Risk

**Fig. 3:**
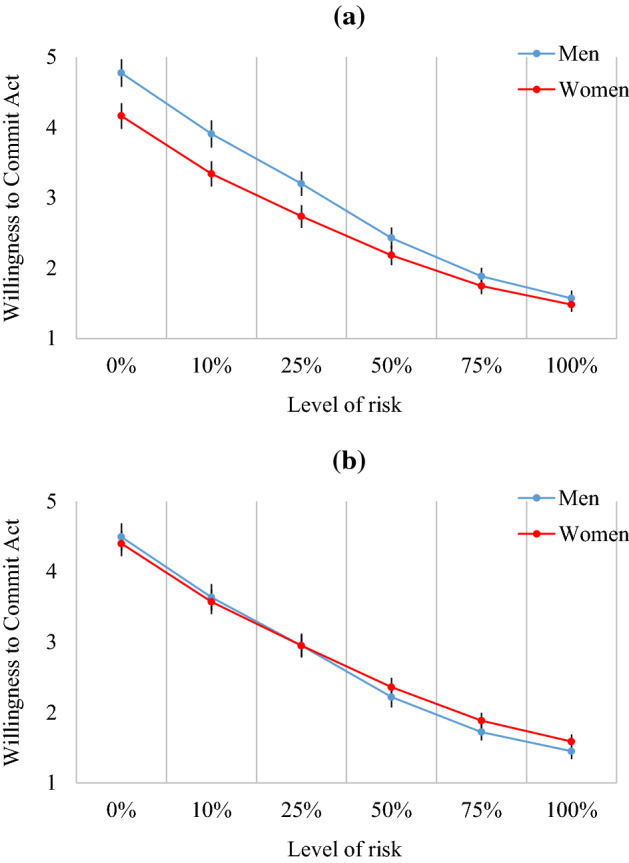
Willingness to commit an act of voyeurism depending on the level of risk involved. Sex differences at low risk are apparent **a**, but disappear when sociosexuality and sexual compulsivity are included as covariates **b**. Error bars = 95% confidence intervals

In order to examine the impact of risk on anticipated voyeuristic action, we ran two-way mixed model ANOVAs using sex and risk-level as factors. We then ran the analysis a second time, but with SOI-R and SCS as covariates to examine how this affected any sex differences present. The initial ANOVA revealed a main effect of sex, *F*(1, 1111) = 14.15, *p* < .001, η_p_^2^ = 0.011 [90% CI = 0.003, 0.026], with men (*M* = 2.96, *SE* = 0.07) reporting a higher willingness to commit an act of voyeurism than women (*M* = 2.61, *SE* = 0.06) regardless of level of risk. However, this effect of sex was minute compared to the substantial main effect of risk, showing a linear downward trend as risk increased, *F*(1, 1111) = 1678.94, *p* < .001, η_p_^2^ = 0.601 [CI = 0.575, 0.626]. Finally, a sex by risk interaction revealed that these linear trends had sex-differentiated slopes, *F*(1, 1111) = 16.40, *p* < .001, η_p_^2^ = 0.014 [CI = 0.005, 0.028]. Planned comparisons revealed that the difference was largest (*d* = 0.27) at 0% risk (0.61 [95% CI = 0.34, 0.88]) and this narrowed as risk approached 50% (0.25 [0.04, 0.45]). Above this level of risk, 95% confidence intervals crossed zero (see Fig. 3a).

When we reran the analysis including SOI-R and SCS as covariates, there was no longer a main effect of sex, *F*(1, 1109) = 0.25, *p* = .62, with men (*M* = 2.74, *SE* = 0.07) reporting a similar willingness to commit an act of exhibitionism as women regardless of the risk involved (*M* = 2.79, *SE* = 0.06). There continued to be a linear effect of risk, *F*(1, 1109) = 25.50, *p* < .001, η_p_^2^ = 0.022 [CI = 0.010, 0.039], with a clear drop at each risk increment (*p* < .001). This linear trend continued to differ by sex, *F*(1, 1110) = 2.57, *p* < .05, η_p_^2^ < 0.01 [CI = 0, 0.009], though this difference was reduced considerably (see Fig. 3b). Planned comparisons revealed that the 95% confidence intervals of the sex difference crossed zero at all levels of risk.

**Fig. 4:**
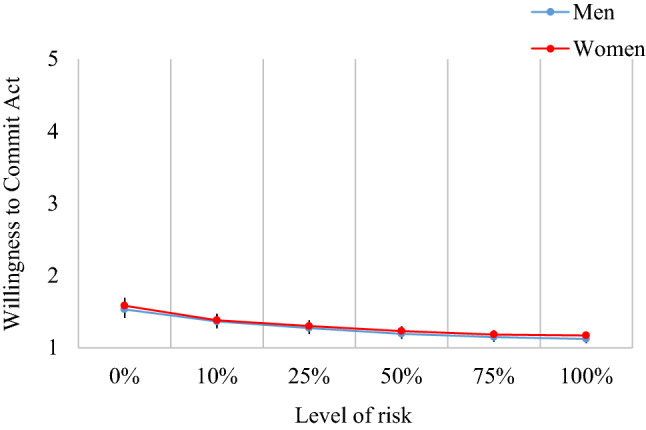
Willingness to commit an act of exhibitionism depending on the level of risk involved. Error bars = 95% confidence intervals

For exhibitionism, there was no overall difference between the sexes, *F*(1, 1111) = 0.47, *p* = .49. As with voyeurism, there was a linear trend of risk, *F*(1, 1111) = 118.41, *p* < .001, η_p_^2^ = 0.096 [CI = 0.070, 0.124], but a lack of risk by sex interaction indicated that this trend was similar for both sexes, *F*(1, 1111) = 0.16, *p* = .98; see Fig. 4. As no sex differences were present, we did not proceed with planned comparisons nor the inclusion of SOI-R and SCS as covariates.

Together, the results of this task provide partial support for H4 and H5. Specifically, while there was a clear sex difference in the willingness to commit a voyeuristic act, which was mediated by a combination of sociosexuality and sexual compulsivity, this was not the case for exhibitionism.

## Discussion

Our investigation into the role of sociosexuality in voyeuristic and exhibitionistic interest yielded four key findings. (1) There were clear sex differences in voyeuristic and exhibitionistic repulsion within the normal population. (2) These differences were partially mediated by the serial combination of sociosexuality and sexual compulsivity. (3) There were sex-specific relationships between sociosexuality and sexual compulsivity when predicting exhibitionistic repulsion. Finally, (4) the sex differences and mediational role of sociosexuality and sexual compulsivity found for repulsion also applied to willingness to commit acts of voyeurism, but not exhibitionism. We now explore these findings in turn.

### Sex Differences in Repulsion within the Normal Population

Most participants reported feelings of repulsion, rather than arousal, when thinking about voyeurism and exhibitionism. Yet, there was still variance among the sample, with some considering these acts either neutral or arousing. These individuals constituted approximately 29.7% and 8.9% of the sample for voyeurism and exhibitionism, respectively. These figures are fairly consistent with literature from other Western cultures (Ahlers et al., 2011; Joyal & Carpentier, 2017) though the proportion was a touch smaller for the latter and larger for the former. Importantly, we also found a sex difference: Men were less repulsed by the idea of voyeurism and exhibitionism, on average, than women were. These differences were large in the case of voyeurism and medium in the case of exhibitionism, which is in line with previous research in other countries (Dawson et al., 2016; Iwawaki & Wilson, 1983; Makanjuola et al., 2008; Oliveira Júnior & Abdo, 2010). When sex differences in mating interests and motivations transcend cultural boundaries and persist across a variety of contexts, this suggests that their development is fairly canalized (Thomas et al., 2020). Thus, it is important to consider what insights evolutionary theory may provide when investigating such differences.

### Sociosexuality and Sexual Compulsivity as Partial Mediators

Using mediation analyses, we found that a combination of sociosexuality and sexual compulsivity accounted for a large portion of the sex difference in voyeuristic (31%) and exhibitionistic (56%) repulsion. These findings complement previous research which has demonstrated that paraphilic interests are linked to sociosexuality and are partially mediated by sexual compulsivity (Dawson et al., 2016).

Uniquely, we used a model that specified a serial relationship between sociosexuality and sexual compulsivity, with the former driving the latter. This prediction was grounded in evolutionary thinking (Buss & Schmitt, 1993; Gangestad & Simpson, 2000) that views sociosexuality as a proxy for mating strategy. From this perspective, the long evolutionary history of sexual asymmetry in the reproductive costs and benefits associated with short-term mating has led men to develop a greater interest in it on average. Indeed, the sex difference in sociosexual desire is among one of the largest found in psychology (*d* = 0.88 in the present study). Thus, one’s sex impacts one’s mating strategy, which in turn leads to the activation of psychological adaptations designed to facilitate that strategy, including increased sexual compulsion and greater arousal at the thought of engaging in spontaneous sexual acts with strangers. Together, these findings have implications for the way we view some courtship disorders. If sex differences in voyeuristic and exhibitionistic interests are a consequence of sex differences in sociosexuality, then men may be better represented among those with extreme versions of such interests (i.e., those with paraphilic disorders) because moderate sex differences in means translate into large sex differences at the extremes (Stewart-Williams & Thomas, 2013a).

An evolutionary approach to this issue also allows us to make predictions about what should influence voyeuristic and exhibitionistic repulsion. For example, if these interests reflect mating strategy, then we would expect them to covary with other factors associated with short-term mating. Thus, an evolutionary account of these phenomena would predict attitudes toward voyeurism to be more relaxed when resources are abundant or in stressful environments where the benefits of paternal investment are lower (Quinlan, 2007; Thomas & Stewart-Williams, 2018). There is also scope to explore the role mating strategy plays in other sex differences, such as responses to visual sexual stimuli. As in the present study, we might expect sociosexuality to mediate this difference and that responses to visual sexual stimuli may moderate interest in voyeuristic or exhibitionistic activity (Rupp & Wallen, 2008).

Of course, a major premise of this study is that sociosexuality is a reliable proxy of short-term mating strategy. This assumption is not unique to our study, and others have used sociosexuality as a proxy for mating effort (e.g., Dawson et al., 2016). We chose the SOI-R as our key measure because it is one of the most widely used and validated measures of the preference for uncommitted sex. Yet, the use of this measure comes with limitations, it forces us to assume that short- and long-term strategies lie on a spectrum rather than being activated separately (Thomas & Stewart-Williams, 2018) and it neglects to measure the time and resources dedicated to mating effort (Albert et al., 2021). Future research could use a wider array of measures to assess whether similar patterns are observed when using different proxies of mating strategy and reproductive success. Other measures of sex drive should also be considered to overcome some of the limitations of the SCS. At its core, the scale captures the extent to which sexual thoughts and behaviors interfere with one’s daily life. While research using the scale demonstrates its usefulness as a measure of individual difference in the general population (e.g., Carvalho et al., 2015; Muise et al., 2013), it is possible that other measures of sex drive would capture more variance. Finally, while we recorded repulsion toward a large array of paraphilia through use of the Paraphilia Scale (Seto et al., 2012), we only sought to explain sex differences in the two for which we had a priori hypotheses. It may be worth considering what evolutionary insights might bring to other paraphilia, unrelated to the early stage of courtship process in the future. Those involving elements of control (e.g., sadism and masochism), for example, may reflect other evolved aspects of our mating psychology such as paternity certainty and mate guarding (Goetz et al., 2008).

### Sex-Specific Patterns of Predictors

We expected both voyeuristic and exhibitionistic repulsion to have similar relationships with sociosexuality and sexual compulsion as they share a common root in their spontaneous and opportunistic approach to courtship. Our sex-specific models revealed that sociosexuality was a positive predictor of repulsion for both acts in men and women alike. However, we did find some variation in the role of sexual compulsivity in predicting exhibitionistic repulsion. For men, sociosexuality predicted unique variance in exhibitionistic repulsion even when controlling for sexual compulsivity, while for women sexual compulsivity fully mediated the effect of sociosexuality. Given our theoretical stance that sexual compulsivity is primarily a product of mating strategy rather than the cause, the results can be interpreted as follows: Men with unrestricted sociosexuality find exhibitionism more appealing for reasons that include but are not limited to their increased drive toward sex. Unrestricted women, in contrast, find exhibitionism more appealing because of their increased sexual compulsivity. Together, this suggests that the way in which mating strategy gives rise to exhibitionistic interest may be more nuanced for men than women, possibly reflecting different selection pressures.

### From Feelings to Action

When participants actually considered committing acts of voyeurism, they were heavily influenced by the risk of getting caught. However, a sex difference also emerged that reached its peak (*d* = 0.27) when risk was negligible. This sex by risk interaction was not found in previous studies, likely due to low power (Rye & Meaney, 2007). This effect size indicates that sex differences in voyeuristic interest decrease when moving from repulsion toward anticipated action. When we controlled for sociosexuality and sexual compulsivity, this sex difference disappeared. Thus, while these two variables only partially mediate repulsion, they fully mediate anticipated action. Put another way, the combination of sociosexuality and sexual compulsivity is only one of several explanatory factors of the sex difference in voyeuristic repulsion, but is the key factor when explaining willingness to engage in a voyeuristic act.

The findings for exhibitionism were markedly different. Sex differences at the level of repulsion were not replicated when examining anticipated action. Essentially, there was a large floor effect with both sexes showing considerably less interest in performing an act of exhibitionism. While a small effect of risk was found, there were no sex differences to explain. Previous research has shown sex differences in general paraphilic behavior to be moderated by sexual compulsivity (Bouchard et al., 2017). Our findings highlight the importance of considering paraphilia separately as this relationship may not generalize across all behaviors, even when they fall within similar domains (i.e., courtship).

Interest in exhibitionistic behavior increases (as do sex differences) when the definition is expanded to include more passive behavior, such as performing sexual acts with a partner in locations where a stranger is likely to see (Joyal & Carpentier, 2017) rather than actively exposing oneself to a stranger. Thus, repeating the task using this context might reveal sex differences. If so, this would suggest that willingness to commit these acts depends on the level of involvement as well as risk. Future research may also want to consider more contemporary exhibitionistic behavior—such as sending a digital photograph of one’s genitalia to an opposite sex stranger (an increasingly common practice; Mandau, 2020).

### Limitations and Conclusion

Because our sample was from the UK, we should exercise caution in generalizing the findings. Sex differences in a number of mating interests and behaviors vary from culture-to-culture depending on local conditions such as pathogen prevalence and sex ratio (Gangestad et al., 2006; Schacht & Borgerhoff Mulder, 2015), though some are more canalized than others (Thomas et al., 2020). If sex differences in paraphilic interest vary then so might the mediational role of sociosexuality. Cultural variation in openness to sensitive sexual topics may also impede our ability to examine cross-cultural consistency in the factors that underpin voyeuristic and exhibitionistic repulsion. Issues of topic sensitivity are also relevant to the present study as they may have caused response bias. We did give our participants the opportunity to tell us whether they were being untruthful during the questionnaire, which we were then able to use to screen the responses, but traditional measures of social desirability may be a better option for future studies (King & Bruner, 2000). A final limitation involves the potential biases introduced into mediation models by using cross-sectional data (Maxwell & Cole, 2007). While we found that the serial combination of SOI-R and SCS accounted for more variance than its opposite, a longitudinal design would provide stronger evidence for a causal serial relationship.

The association of voyeuristic and exhibitionistic interest with sociosexuality also raises questions about the status of these disorders as pathologies. The harmful dysfunction analysis of psychopathology suggests that there must be both a malfunction of an evolved adaptation in addition to a value laden assessment of the phenomenon as undesirable (i.e., harmful to others) for it to be pathological (Kennair, 2011; Wakefield, 1999). Different aspects of our sexual psychology have been considered to show signs of pathology (e.g., negative post-coital emotion; Fernandes et al., 2016). However, an analysis based on evolved sex differences suggests that sometimes these phenomena show signs of adaptation and may therefore not be true pathology, despite being aversive (Fernandes et al., 2016). A similar perspective could be applied to voyeurism and exhibitionism. It is important to note that we are not addressing pathology or disorder as such. However, we are considering the normal sexual psychological mechanisms that underlie both disordered behavior as well as normal variance in individual differences in sexual psychology (see Nesse, 2019 on the problem of considering the disorder an adaptation). The current paper addresses exhibitionism and voyeurism within normal range as nonpathology and hypothesizes that full-blown disorders represent, in part, extrapolations of this at nonadaptive levels likely exacerbated by some of the comorbidities that typically accompany these disorders such as antisocial personality and substance-related disorders (Marshall, 2007).

In conclusion, this study replicated the well-established finding that the sexes differ, on average, in how repulsed they are by voyeurism and exhibitionism and that sociosexuality and sexual compulsivity mediate these differences. Using an evolutionary perspective, which sees sociosexuality as a proxy for mating strategy, we predicted and found serial relationships between these mediators. We also found sex-specific patterns underlying exhibitionistic, but not voyeuristic interest, highlighting the importance of examining the sexes separately. Sociosexuality and sexual compulsivity also accounted for sex differences in the intention to commit voyeurism, but not exhibitionism, which appears to be universally unappealing perhaps because of its more “active” involvement. Nonetheless, sociosexuality appears an important predictor in exhibitionistic and voyeuristic repulsion. To the extent that sociosexuality acts a proxy for mating strategy, these results have implications for how we view courtship disorders, suggesting they are extreme nonfunctional versions of desires and behaviors manifested as part of an evolved short-term mating strategy. Thus, sex differences at the disorder level may be symptomatic of the moderate sex differences in inclination toward short-term sex.

## Data Availability

Data are available from the first author upon request.

## Footnotes

1 One of the goals of this research was to understand individual differences in voyeuristic and exhibitionistic arousal. In reality, most participants typically report feeling repulsed at the idea of voyeurism and exhibitionism. Thus, we use the term “repulsion” throughout this work.

2 In the present study, we focus on this sexual compulsivity measure alone and so, for accuracy, refer to sexual compulsivity from here on in.

3 This question provided a brief method of screening participants. Four was chosen as a cutoff point as it struck a conservative balance; it resulted in 15 participants being excluded from the sample compared to 48 for a cutoff point of 5. The mediational models were qualitatively similar when all participants were included in the analyses.

4 At the request of the reviewers, we also ran both models with SOI-R and SCS reversed. All indirect effects were present in this revised models. However, the serial pathway accounted for much less variance. For the voyeuristic model, the indirect serial pathway from SCS SOIR (− .03; 95% CI = − .04, − .01) accounted for 4% of the variance (vs. 8%). In the exhibitionistic model, the SCS SOIR pathway (− .02; 95% CI = − .03, − .01) accounted for 6% of the variance (vs. 16%).
